# Differential target multiplexed spinal cord stimulation using a paddle-type lead placed at the appropriate site for neuropathic pain after spinal cord injury in patients with past spinal surgical histories: study protocol for an exploratory clinical trial

**DOI:** 10.1186/s13063-023-07433-7

**Published:** 2023-06-13

**Authors:** Takafumi Tanei, Satoshi Maesawa, Yusuke Nishimura, Yoshitaka Nagashima, Tomotaka Ishizaki, Masahiko Ando, Yachiyo Kuwatsuka, Atsushi Hashizume, Shimon Kurasawa, Ryuta Saito

**Affiliations:** 1 Department of Neurosurgery, Nagoya University Graduate School of Medicine, 65 Tsurumai-cho, Showa-ku, Nagoya, Aichi 466-8550 Japan; 2 Department of Advanced Medicine, Nagoya University Graduate School of Medicine, 65 Tsurumai-cho, Showa-ku, Nagoya, Aichi 466-8550 Japan; 3 Department of Clinical Research Education, Nagoya University Graduate School of Medicine, 65 Tsurumai-cho, Showa-ku, Nagoya, Aichi 466-8550 Japan

**Keywords:** Spinal cord stimulation, Spinal cord injury, Neuropathic pain, Paddle lead, Differential target multiplexed

## Abstract

**Background:**

Neuropathic pain after spinal cord injury (SCI), both traumatic and non-traumatic, is refractory to various treatments. Spinal cord stimulation (SCS) is one of the neuromodulation therapies for neuropathic pain, although SCS has insufficient efficacy for neuropathic pain after SCI. The reasons are presumed to be inappropriate locations of SCS leads and conventional tonic stimulation itself does not have a sufficient analgesic effect for the pain. In patients with past spinal surgical histories, the cylinder-type leads are likely to be placed on the caudal side of the SCI because of surgical adhesions. Differential target multiplexed (DTM) stimulation is one of the latest new stimulation patterns that is superior to conventional stimulation.

**Methods:**

A single-center, open-label, randomized, two-way crossover trial is planned to investigate the efficacy of SCS using DTM stimulation placing a paddle lead at the appropriate site for neuropathic pain after SCI in patients with spinal surgical histories. The paddle-type lead delivers energy more efficiently than a cylinder-type lead. This study consists of two steps: SCS trial (first step) and SCS system implantation (second step). The primary outcome is rates of achieving pain improvement with more than 33% reduction 3 months after SCS system implantation. The secondary outcomes are to be evaluated as follows: (1) effectiveness of DTM and tonic stimulations during the SCS trial; (2) changes of assessment items from 1 to 24 months; (3) relationships between the result of the SCS trial and the effects 3 months after SCS system implantation; (4) preoperative factors associated with a long-term effect, defined as continuing for more than 12 months; and (5) whether gait function improves from 1 to 24 months.

**Discussion:**

A paddle-type lead placed on the rostral side of SCI and using DTM stimulation may provide significant pain relief for patients with intractable neuropathic pain after SCI in patients with past spinal surgical histories.

**Trial registration:**

Japan Registry of Clinical Trials (jRCT) jRCT 1042220093. Registered on 21 November 2022, and last modified on 6 January 2023. jRCT is approved as a member of the Primary Registry Network of WHO ICTRP.

## Background

Spinal cord injury (SCI) induces paralysis and other dysfunctions, typically pain symptoms, in 30–80% of SCI patients [1–4]. Pain symptoms after SCI consist mainly of musculoskeletal and/or neuropathic pain [5, 6]. The pain after SCI impairs quality of life, and lost productivity and the cost of treatment pose a heavy economic burden for patients and their families, as well as society [2–4]. The damaged spinothalamic tracts after SCI are related to enhanced neuronal excitability and reduced descending pain inhibition, leading to chronic central neuropathic pain [7–9]. On a cellular level, microglial cells and astrocytes are activated in the early phase after SCI to remove debris and damaged cells [10]. These glial cells can be persistently activated and release several chemicals, which contribute to the development of central sensitization and neuropathic pain. In addition, hypersensitive neurons in the dorsal column of the spinal cord mediate pain secondary to increased aberrant background activity and altered sodium channel currents. Non-traumatic causes, such as spinal tumors, vascular malformations, or chronic spinal compression, may also induce spinal cord parenchymal damage. Such spinal damage can also induce neuropathic pain through a similar pathogenic mechanism as traumatic SCI. Various treatments have been used for the neuropathic pain after SCI, including medication, rehabilitation, psychotherapy, and neuromodulation therapy, but these treatment methods do not provide sufficient pain relief [11–14].

Spinal cord stimulation (SCS) is one of the neuromodulation therapies, and it has been used for decades to treat chronic neuropathic pain [15, 16]. The conventional paresthesia-based SCS uses tonic stimulation that induces a sense of paresthesia [17, 18]. It is essential that the elicited paresthesia overlaps the painful area to ameliorate pain symptoms [19]. Conventional SCS for neuropathic pain after SCI has success rates of approximately 30% [13, 14, 20]. The success rates are lower than of SCS for failed back surgery pain syndromes or peripheral neuropathic pain. Therefore, the efficacy of SCS for neuropathic pain after SCI is controversial, and current treatment guidelines do not recommend it [20–23]. The reasons are assumed to be due mainly to two factors: SCS leads may be not placed at an appropriate location, and conventional tonic stimulation itself may have an insufficient analgesic effect for neuropathic pain after SCI.

In patients with past spinal surgical histories, the cylinder-type leads are likely to be placed on the caudal side of the SCI because of adhesions around previous surgeries. The pain relief mechanism of SCS is based on the gate control theory [24, 25]. According to that theory, an appropriate SCS lead location is not on the caudal side, but on the rostral side of the SCI, which induces activation of spinal GABAergic interneurons in the dorsal horn and descending pain-inhibitory pathways [24, 25]. The first clinical question is whether SCS with a paddle-type lead placed on the rostral side of the SCI provides more pain relief. A paddle-type lead device can deliver energy more efficiently than a cylinder-type lead device, although laminectomy is necessary for paddle-type lead placement [26–28].

Recently, several new stimulation patterns without paresthesia have been developed [29–32]. Differential target multiplexed (DTM) stimulation (Medtronic Inc., Minneapolis, MN, USA) is one of the latest paresthesia-free SCS patterns, and it has the potential to be superior to conventional tonic stimulation [33]. DTM stimulation uses multiple electrical signals, and it has been shown to modulate gene expressions in the spinal cord at the site of stimulation [34–36]. Modulation of glial cells and neurons and rebalancing their interactions are considered to be among the mechanisms of pain relief by DTM stimulation [34–36]. Activity and modulation of glial cells are key factors in both neuropathic pain after SCI and DTM stimulation [10, 34–36]. The second clinical question is whether SCS using DTM stimulation provides more pain relief than conventional tonic stimulation for neuropathic pain after SCI.

We hypothesized that DTM stimulation using a paddle-type lead placed on the rostral side of the SCI provides sufficient pain relief for patients with intractable neuropathic pain after SCI with past spinal surgical histories. In fact, we have reported three cases of good outcomes using this method [26]. The aim of this study is to evaluate this hypothesis.

## Methods/design

### Study design

This is a prospective, single-center, open-label, randomized, two-way crossover, exploratory trial.

### Patient population

Patients are selected based on the inclusion and exclusion criteria described below. The main criterion for enrollment is intractable neuropathic pain after traumatic or non-traumatic SCI with histories of spinal surgery at the site where the SCS lead will pass or be placed. Patients will be recruited from new patient from standard clinical practice at Nagoya University Hospital.

### Inclusion criteria

- ▪ Patients with intractable neuropathic pain after SCI resistance to drug treatments
- ▪ Patients with histories of spinal surgery at the site where the SCS lead will pass or be placed
- ▪ 18 years of age or older
- ▪ Visual analog scale (VAS) score for pain greater than 40 points
- ▪ Patients classified as Frankel grade B–E
- ▪ Patients in whom a paddle-shaped lead can be placed on the rostral side of the spinal cord lesion
- ▪ Patients who have given written, informed consent.

### Exclusion criteria

- ▪ Patients for whom local and general anesthesia cannot be performed
- ▪ Patients classified as Frankel grade A
- ▪ Patients receiving anti-cancer therapy
- ▪ Patients with a history of drug abuse
- ▪ Patients at high risk for surgery, such as patients with unstable angina pectoris and with end-stage liver disease presenting with hepatic encephalopathy
- ▪ Patients with diabetes mellitus who are not well controlled (HbA1c ≥9%)
- ▪ Patients with serious complications (liver disease, kidney disease, heart disease, lung disease, blood disease, brain disease, etc.)
- ▪ Pregnant or potentially pregnant patients
- ▪ Patients considered inappropriate by a head of research or investigator

### Who will take informed consent?

Potential participants will be identified from patients who visit Nagoya University Hospital. After assessment by clinical research physicians regarding inclusion and exclusion criteria, the study information will be given to potential participants. Written and verbal informed consent will be obtained. The right of a participant to refuse to participate in this trial without giving reasons for the decision will be respected. This trial does not involve collecting biological specimens for storage.

### Study procedures

**Fig. 1:**
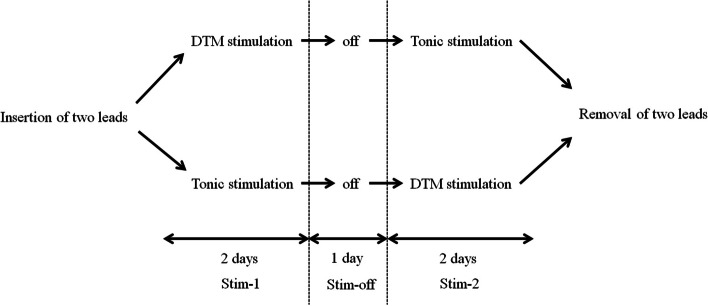
The order of stimulation patterns. The order of stimulation patterns of DTM or tonic is randomly assigned to two courses. One course is DTM stimulation (Stim-1), then tonic stimulation (Stim-2). The other course is in reverse order. Each stimulation pattern is performed for two days. The stimulation off period is set as one day between Stim-1 and Stim-2 (Stim-off)

This trial consists of a two-step procedure. The first step is an SCS trial, and the second step is SCS system implantation. The lead locations for the SCS trial and SCS system implantation are the sides caudal and rostral to the SCI, respectively. In the SCS trial, two cylinder-type leads (Model 977A190; Medtronic Inc., Minneapolis, MN, USA) are to be inserted on the caudal side of the SCI under local anesthesia, so that the lead does not pass through the SCI. Once inserted, the two leads are directly sutured to the skin at the puncture sites without skin incision. The order of stimulation patterns of DTM or tonic is randomly assigned to two courses evenly (Fig. 1). One course is DTM stimulation (Stim-1), then tonic stimulation (Stim-2), and the other course is in reverse order. Each stimulation pattern is performed for two days. The stimulation off period is set as one day between Stim-1 and Stim-2 to washout previous stimulation effects (Stim-off).

**Fig. 2:**
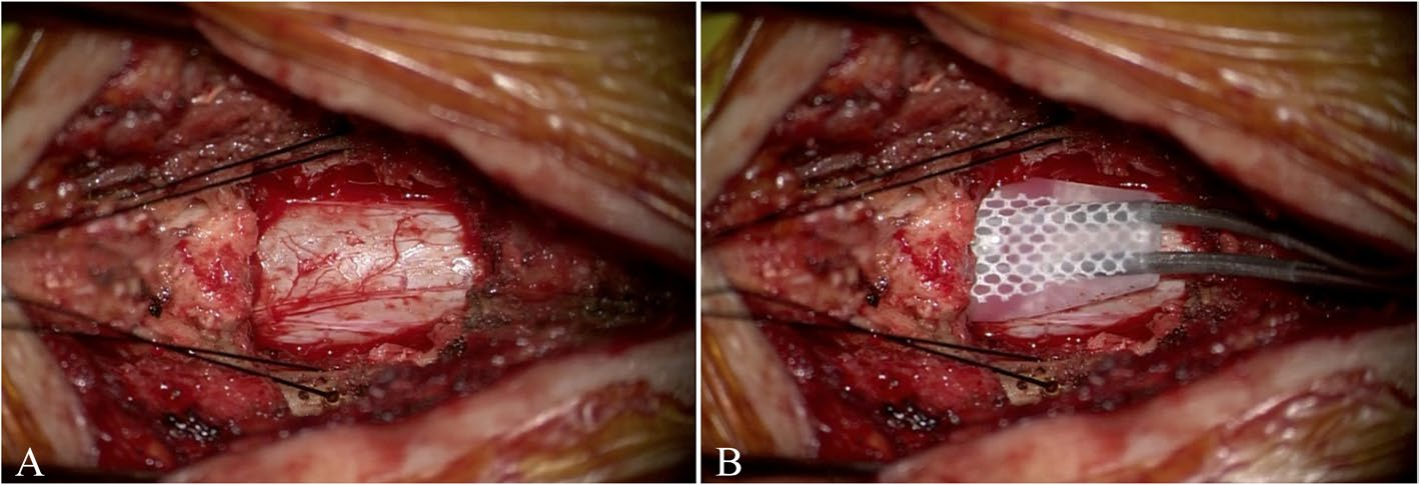
Intraoperative photograph of a paddle-type lead placing. **A** The spinal dura matter is exposed by laminectomy under general anesthesia. **B** A paddle-type lead is inserted at the epidural space

After the SCS trial, the inserted leads are removed whether or not analgesic effects are obtained. In cases of an effective SCS trial or the patient agrees to implantation of an SCS system even after a non-effective SCS trial, the second step follows. With an interval of 1 month from the SCS trial, a paddle-type lead is placed on the rostral side of the SCI by laminectomy under general anesthesia (Fig. 2). The location of the lead is confirmed by intraoperative X-ray and motor-evoked potential monitoring. At the same time, an implantable pulse generator (IPG) (Intellis; Medtronic Inc.) is implanted on the hip, and it is connected to the paddle-type lead. After the SCS implantation, an effective stimulation pattern that showed efficacy in the SCS trial is started, and the analgesic effects are evaluated. In the absence of analgesic effects in the previous SCS trial, DTM stimulation is tried.

### Setting stimulation parameters

Tonic stimulation delivers mild electrical pulses and elicits paresthesia. Stimulation parameters include frequency, pulse width, and voltage. Frequency (10–100 Hz) and pulse width (40–300 µs) are set by clinical research physicians so that the patient feels comfortable with paresthesia. The voltage is adjusted by the patient using a remote controller to the intensity level of eliciting comfortable paresthesia for 2 days.

DTM stimulation delivers multiple electrical signals and stimulates multiple locations without paresthesia. DTM stimulation consists of one stimulation signal on the upper side of the lead, called the base program, and multiple stimulation signals on the lower side of the lead, called the prime program. For the base program, frequency (50 Hz), pulse width (200 µs), and voltage (approximately 70% of the paresthesia threshold) are set. For the prime program, frequency (300 Hz), pulse width (170 µs), and voltage (approximately 65% of the paresthesia threshold) are set. DTM stimulation delivers electrical signals from one base and three prime programs. DTM stimulation is set by clinical research physicians; the stimulation continues for 2 days, and the patient does not adjust the stimulation parameters.

### Criteria for discontinuing or modifying allocated interventions

Any patients requesting to end their participation in the study can be withdrawn from the study regardless of the stage they have reached in the study process. Patients do not have to provide the reason of withdrawal. Patients found to be pregnant or those judged ineligible to continue participating in the study by the investigators will also be withdrawn from the study.

### Strategies to improve adherence to interventions

All treatments will be administered to participants during their stay in the hospital by attending surgeons. Therefore participants’ adherence to interventions is assured.

### Relevant concomitant care permitted or prohibited during the trial

All other treatments will be allowed.

### Provisions for post-trial care

All patients who will suffer harm from trial participation will be covered by the Japanese public healthcare system.

### Clinical assessments

At entry, clinical research physicians obtain information from patients regarding age, sex, past history, medications, cause of SCI (traumatic or non-traumatic), level of SCI (cervical, thoracic, lumbar), location of pain (arm, leg, lower back, back, chest), degree of paralysis (none, mild, moderate, severe), sensory disturbance (hypoesthesia, allodynia, numbness), duration of disease, and Frankel grade (B-E). During the SCS trial, the degree of pain is evaluated using a VAS according to the plan (Table 1). After SCS system implantation, assessment items are evaluated according to the plan, including degree of improvement of pain relief, mental health, comprehensive health, and gait function (Table 1). Six months after SCS system implantation, if no improvement of any of the assessment items is seen, removal of the implanted SCS system would be considered.

**Table 1:**
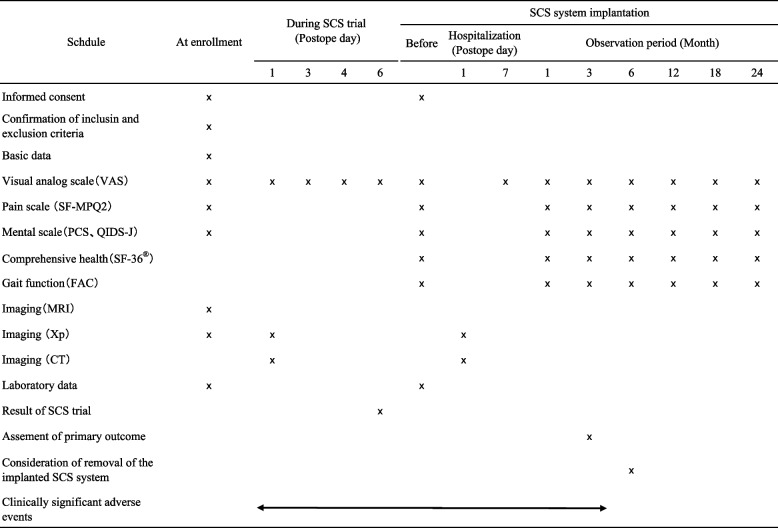
Summary of observations, assessment items, and schedule of assessments

*VAS* visual analog scale, *SF-MPQ-2* short-form McGill pain questionnaire-2, *PCS* pain catastrophizing scale, *QIDS-J* quick inventory of depressive symptomatology, *SF-36* short-form 36-item health survey, *FAC* functional ambulation categories, *MRI* magnetic resonance image, *Xp* X-ray picture, *CT* computed tomography, *SCS* spinal cord stimulation

### Assessment items

Assessments of pain relief are performed by a VAS and the short-form McGill pain questionnaire-2 (SF-MPQ-2). Assessments of mental health and comprehensive health are performed by the pain catastrophizing scale (PCS), quick inventory of depressive symptomatology (QIDS-J), and the Short-Form 36-item health survey (SF-36^®^). Assessment of gait function is performed by functional ambulation category (FAC).

### Imaging evaluation

The location of the spinal cord lesion is confirmed by magnetic resonance imaging (MRI) taken before the initial spinal surgery for SCI. Before the SCS trial, whole spinal MRI is performed to rule out any other abnormal findings, and whole spinal X-rays are also performed to confirm the location and type of fixation devices implanted in previous spinal surgeries. After the SCS trial and SCS system implantation, spinal X-rays and computed tomography (CT) are performed to confirm the location of the SCS leads and to rule out postoperative complications.

### Primary outcome

Pain improvement is defined as more than a 33% reduction in the VAS score. The primary outcome is rates of achieving pain improvement at 3 months after SCS system implantation.

### Secondary outcomes

The secondary outcomes are as follows: (1) evaluation of effectiveness of DTM and tonic stimulations in the SCS trial; (2) evaluation of changes of assessment scores, VAS, SF-MPQ-2, PCS, QIDS-J, and SF-36^®^, at 1, 3, 6, 12, 18, and 24 months from SCS system implantation; (3) relationship between the result of the SCS trial and the effect 3 months after SCS system implantation; (4) evaluation of preoperative factors associated with a long-term effect, defined as continuing for more than 12 months; (5) evaluation whether there is improvement of motor function using the gait function scale (FAC) at 1, 3, 6, 12, 18, and 24 months from SCS system implantation; and (6) adverse events related to this study 3 months after SCS system implantation.

### Sample size

There is no reference clinical data for the efficacy of DTM stimulation using a paddle-type lead placed on the rostral side of the SCI because there is only one case report [26]. Therefore, the sample size is not able to determine on statistical assumptions. Assume that there are 2 to 4 cases who meet the inclusion criteria of this study in a year. The sample size is set 10 as the number that can be achieved in 3 to 5 years.

### Data management

Registration, randomization, and data collection are performed using an electronic data capture (EDC) system. Randomization is performed centrally through the web-based system with a minimization procedure. The allocation sequence using the web-based system is generated by YK and MA belonging to the Department of Advanced Medicine of Nagoya University Hospital. Enrolling participants and assignment to interventions are performed by TT. Data will be collected via research electronic data capture (REDCap). Statistical analyses are planned at the data center (Department of Advanced Medicine, Nagoya University Hospital).

### Concealment mechanism

The results of the allocation will be shown via the interactive web response system.

### Assignment of interventions: blinding

#### Who will be blinded?

Trial participants will be blinded to which group they are assigned.

#### Procedure for unblinding if needed

Not applicable. The study design is open label with only data analysts being blinded so unblinding will not occur.

### Plans to promote participant retention and complete follow-up

Medical interviews and adjustments of SCS will be booked for all patients.

### Confidentiality

The form used to code patients will be stored in a locked cabinet with logged access only available to the researchers and administrators responsible for the study.

### Plans for collection, laboratory evaluation, and storage of biological specimens for genetic or molecular analysis in this trial/future use

Not applicable.

### Statistical analysis

The primary outcome will be estimated as the probability of patients achieving more than 33% pain improvement on the VAS at 3 months after SCS system implantation. The secondary outcome analyses are as follows:

1. A logistic regression analysis using stimulation timing (Stim-1, Stim-2), stimulation pattern (tonic, DTM), pre-VAS values for each stimulation, and interaction between stimulation timing and stimulation pattern as explanatory variables. The carryover effect is examined by testing the interaction term at a significance level of 10%. If a carryover effect is observed, an additional analysis will be performed using only Stim-1.
2. Adjusted means and 95% confidence intervals (CIs) of the rate of change or amount of change for each item (VAS, SF-MPQ-2, PCS, QIDS-J, and SF-36^®^) are calculated for each evaluation time point including pre-implantation and post 1, 3, 6, 12, 18, and 24 months using linear mixed models with time points as fixed effects. The amount of change of each item at each time point is compared after using the Dunnet-Hsu correction for multiple comparisons.
3. For the patients undergoing implantation, logistic regression will be performed to investigate the relationship between the efficacy of the SCS trial (DTM) and the effectiveness for 33% improvement on the VAS at 3 months after implantation.
4. Preoperative factors associated with long-term effects are examined using logistic regression analysis as explanatory variables for the following items: sex, age (<60 years vs. ≥60 years), disease duration, pain site (arm, leg, lower back, back, chest), level of SCI (cervical, thoracic, lumbar), paralysis (none, mild, moderate, severe), sensory disturbance (hypoesthesia, allodynia, numbness), and Frankel classification (B, C, D, E).
5. Probability of gait improvement is estimated, defining an improvement as 1 point or more in the FAC category. The probability of improved cases and the 95% CIs will be calculated at each evaluation time point.

Safety analysis will be performed by tabulating the number and probability of each event.

### Interim analyses

Interim analyses are not planned.

### Methods in analysis to handle protocol non-adherence and any statistical methods to handle missing data

No statistical methods will be used to compensate for missing data.

### Plans to give access to the full protocol, participant-level data, and statistical code

Details of the full protocol, participant-level data, or statistical code will not be publicly available. Unpublished data will be made available upon reasonable request to the corresponding author of the publication.

### Oversight and monitoring

#### Composition of the coordinating center and trial screening committee

Nagoya University will serve as the coordinating center. Only the investigators and members of the data center will have access to the anonymized data in REDCap.

#### Composition of the data monitoring committee, its role and reporting structure

Two participating researchers at Nagoya University Hospital will monitor the data. They have the responsibility of verifying patients’ eligibility, written informed consent, compliance with the protocol, and accuracy of the data in REDCap.

#### Adverse event reporting and harms

Researchers will immediately report serious adverse events associated with the trial to the chief investigator. Then, the chief investigator will report serious adverse events to the director of the hospital and the principal investigator. Data about all serious adverse events will also be collected in REDCap.

#### Frequency and plans for auditing trial conduct

During the study period, monitoring will be carried out by the monitoring staffs to ensure that the study is conducted properly. Monitoring staffs are SM and YN, and they are member of the trial team. The monitoring will be conducted by visits, e-mail, etc. at an appropriate frequency. Check the following items: (1) consent acquisition, (2) conducted eligibility, (3) observance of the study protocol, (4) presence or absence of diseases, (5) consistency between source documents and case reports, (6) confirmation of serious illness, (7) clinical study procedures, and (8) storage status of documents.

#### Plans for communicating important protocol amendments to relevant parties

Any protocol modifications will be reviewed by the Certified Review Board of Nagoya University Graduate School of Medicine and then registered at jRCT. All relevant information will be shared among the researchers.

#### Dissemination plans

The results of this study will be published in a peer-reviewed journal and presented at national and international medical congresses.

## Discussion

In general SCS procedures, the SCS lead is placed at the same position in the SCS trial and in SCS system implantation. The appropriate SCS lead position for achieving pain relief is the rostral side of the SCI. However, in patients with past spinal surgical histories, adhesions around past spinal surgeries make it difficult to pass the cylinder-type leads through the SCI site. On the other hand, paddle-type lead placement is more invasive than insertion of cylinder-type leads because it requires laminectomy under general anesthesia. Therefore, an SCS trial using a paddle-type lead is not reasonable. In this study protocol, the SCS trial is planned using cylinder-type leads placed on the caudal side of the SCI. The SCS lead positions are not theoretically appropriate on that side and may not show sufficient pain relief effects. The reasons for performing this SCS trial are as follows. The first reason is to avoid the risk of bleeding or tissue damage by passing the cylinder-type leads through the SCI site. The second reason is to assess whether the result of the SCS trial with caudal stimulation of the SCI is a predictor of the long-term effects of SCS implantation with rostral stimulation of the SCI. The last reason is to provide patients time to decide on permanent SCS system implantation.

The cutoff line for determining whether a treatment is a success or not is important. The 30% to 40% range in pain reduction is widely used as a standard for clinically significant improvement. Hanley et al analyzed the relationship of pain intensity and meaningful change in pain for chronic pain after SCI and amputations [37]. They concluded that a 33% decrease in pain was a reasonable standard for meaningful change for chronic pain after SCI, and 30% may be low. Based on the report, the cutoff line for the primary outcome is set as more than 33% improvement in pain relief in this study. In assessing the effects of multiple stimulation patterns during an SCS trial, there are two main biases. One is the order of stimulation, and the other is the residual effect of previous stimulation. To reduce the former bias, the SCS trial is randomly assigned as DTM and tonic stimulation, followed by their crossover. To reduce the latter bias, a 1-day off period after a previous stimulation is set to wash out the residual effect.

## Trial status

This manuscript is based on the protocol (version 2, last updated on October 5, 2022). The first patient will be recruited in 2023. Recruitment will be completed by March 2028

## Data Availability

Any data required to support the protocol can be supplied on request.

## Abbreviations

CIs: Confidence intervals
CT: Computed tomography
DTM: Differential target multiplexed
EDC: Electronic data capture
FAC: Functional ambulation categories
IPG: Implantable pulse generator
jRCT: Japan Registry of Clinical Trials
MRI: Magnetic resonance image
PCS: Pain catastrophizing scale
QIDS-J: Quick inventory of depressive symptomatology
REDCap: Research electronic data capture
SCI: Spinal cord injury
SCS: Spinal cord stimulation
SF-MPQ-2: Short-form McGill pain questionnaire-2
SF-36^®^: Short-Form 36-item Health Survey
VAS: Visual analog scale
